# Biomedical Mutation Analysis (BMA): A software tool for analyzing mutations associated with antiviral resistance

**DOI:** 10.12688/f1000research.8740.2

**Published:** 2016-08-02

**Authors:** Karina Salvatierra, Hector Florez

**Affiliations:** 1 Faculty of Exact, Chemical and Natural Sciences, Universidad Nacional de Misiones, Posadas, Argentina; 2 Faculty of Technology, Universidad Distrital Francisco José de Caldas, Bogotá, Colombia

**Keywords:** Hepatitis C virus, mutations, resistance, information system

## Abstract

**Introduction:** Hepatitis C virus (HCV) is considered a major public health problem, with 200 million people infected worldwide. The treatment for HCV chronic infection with pegylated interferon alpha plus ribavirin inhibitors is unspecific; consequently, the treatment is effective in only 50% of patients infected. This has prompted the development of direct-acting antivirals (DAA) that target virus proteins. These DAA have demonstrated a potent effect *in vitro* and *in vivo*; however, virus mutations associated with the development of resistance have been described.

**Objective**: To design and develop an online information system for detecting mutations in amino acids known to be implicated in resistance to DAA.

**Materials and methods**:    We have used computer applications, technological tools, standard languages, infrastructure systems and algorithms, to analyze positions associated with resistance to DAA for the NS3, NS5A, and NS5B genes of HCV.

**Results**: We have designed and developed an online information system named Biomedical Mutation Analysis (BMA), which allows users to calculate changes in nucleotide and amino acid sequences for each selected sequence from conventional Sanger and cloning sequencing using a graphical interface.

**Conclusion**: BMA quickly, easily and effectively analyzes mutations, including complete documentation and examples. Furthermore, the development of different visualization techniques allows proper interpretation and understanding of the results.

The data obtained using BMA will be useful for the assessment and surveillance of HCV resistance to new antivirals, and for the treatment regimens by selecting those DAA to which the virus is not resistant, avoiding unnecessary treatment failures. The software is available at: http://bma.itiud.org.

## Introduction

Chronic hepatitis C infection is caused by the hepatitis C virus (HCV) and affects an estimated 200 million people worldwide ^1, 2^. Transmission occurs by percutaneous exposure through blood products. The major risk factors for HCV infection are parenteral exposure, and needle sharing among intravenous drug users. In addition, hemodialysis patients are at risk of contracting an HCV infection ^3– 5^.

Historically, HCV drug therapy has depended on interferon-α and ribavirin and the effectiveness of this combination therapy are primarily determined by the HCV genotype ^6^. The advent of direct-acting antiviral agents (DAA) has paved the way for a new era for the treatment of HCV infection. The most important contribution in their development primarily target protease NS3, protein NS5A or NS5B RNA-dependent RNA polymerase ^7^. However, because the emergence of resistant viral variants, DAA is one of the factors to be taken into account in the treatment ^8^. Antiviral capacity may be limited by the ability of the virus to develop resistance to new antivirals ^9^. Resistance mutations to DAA have been observed both *in vitro* and *in vivo* ^10– 12^. In addition, people infected with HCV, who are left untreated, can develop natural viral variants harboring resistance mutations. Current data indicates pre-existing mutations to NS3 protease inhibitors, NS5A inhibitors, and non-nucleoside inhibitors of NS5B polymerase in 7.7%, 16.2% and 22.5%, of infected patients ^13– 16^. Probably these viral variants contribute to the selection of resistance to DAA during the initial weeks of monotherapy ^17– 20^.

Using DAA implies the possibility of selection of resistant variants. Antiviral resistance results from amino acid substitutions that produce conformational changes that interfere with drug-target interaction. These mutations typically involve a biological cost, and viruses carrying these mutations are found in smaller numbers than wild-type viruses; however, they can be positively selected during therapy ^21^.

Genetic variability affects the response to old and new therapies. It is therefore important to determine mutations of resistance to antiviral drugs.

There is an increasing need to develop bioinformatic tools to analyze the rapidly growing amount of nucleotide and amino acid sequence data in different organisms such as viruses. An important task in bioinformatics is the provisioning of data and tools in a simple manner for users to locate and use. Sequencing generates large amounts of data that need to be analyzed. Advances in information technology have stimulated the development of new computer applications and algorithms for data analysis, and computer visualization tools for the representation of variation patterns. The analysis of mutations is important to understand antiviral resistance and to understand the functions of different proteins. The aim of this study was to develop an online information system named Biomedical Mutation Analysis (BMA), which allows users to calculate changes in nucleotide and amino acid sequences for each selected sequence through a graphical interface.

## Materials and methods

To create the online information system, we used different standard tools, languages, and infrastructure systems. BMA was designed using the Unified Modeling Language (UML) ^23^, which allows describing the system following the Object Oriented Paradigm. Regarding the development of BMA, we used PHP language version 5.3.29 ( https://secure.php.net/), which is supported by Apache software version 2.4.7 ( http://www.apache.org/) as the application server. For the front end of BMA, we used Bootstrap version 3.3.6 ( http://getbootstrap.com/), which is the most popular HTML, CSS, and JavaScript framework for developing responsive web projects. BMA also has some features based on JavaScript language supported by JQuery version 1.12.3 ( https://jquery.com/), which is a JavaScript library that facilitates some specific JavaScript functionalities. BMA provides three different outputs, where two of them use additional support. The former result is a report generated as a pdf file, which is built using ezpdf version 0.0.9 ( https://github.com/rebuy-de/ezpdf), which is a library that supports the creation of pdf files. The latter result is a force-directed graph, which is created using D3 (Data-Driven Documents) version 3.5.16 ( https://d3js.org/), which is an online JavaScript library that helps to deploy data using fancy visualizations.

BMA stores all information related to the mutation analyses in one database supported by MySql version 5.7.12 ( https://www.mysql.com/), which is a relational database management system. The database includes the entities and relationships required for handling all information related to the proposed mutation analyses. The database is manipulated through project phpMyAdmin version 4.3.11 ( https://www.phpmyadmin.net/), which is software written in PHP intended to handle the administration of data stored in MySql databases.

The database was designed using the tool MySql Workbench version 6.3 ( https://www.mysql.com/products/workbench/), while the online system was developed using the tool Eclipse PHP version 3.7.0 ( https://eclipse.org/pdt/). BMA is hosted in a Linux Server debian distribution version 8.4, which includes Apache, MySql, and phpMyAdmin for the right operation of BMA.

All software, frameworks, and libraries used in the design and development of BMA have a GNU General Public License (GNU GPL) ( http://www.gnu.org/licenses/licenses.en.html), which implies that BMA was completely created using free software.

We used the nucleotide sequence of genes NS3, NS5A and NS5B of Con1 isolated HCV genotype 1b (accession number: AJ238799), extracted from GenBank ( www.ncbi.nlm.nih.gov/genbank/) as a reference sequence.

A compilation of resistance mutations previously described *in vivo* and *in vitro* in the literature for the genes NS3, NS5A and NS5B of the HCV were used for computing the number and type of amino acid variants at the corresponding positions associated with resistance to DAA ^24, 25^.

## Results

**Figure 1.**
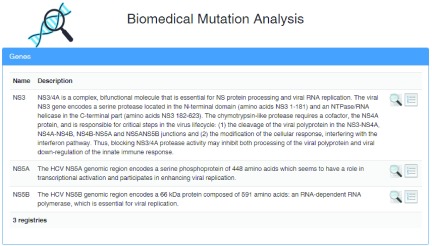
List of available genes for HCV.

**Figure 2.**
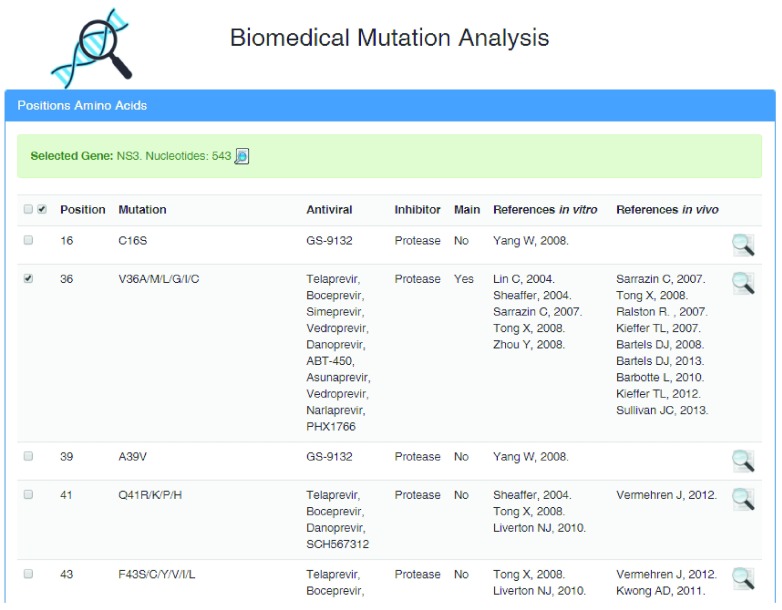
List of positions (5 of 26) for the gene NS3.

**Figure 3.**
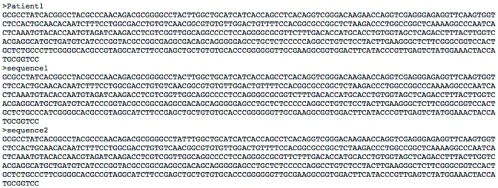
Patient file format.

**Figure 4.**
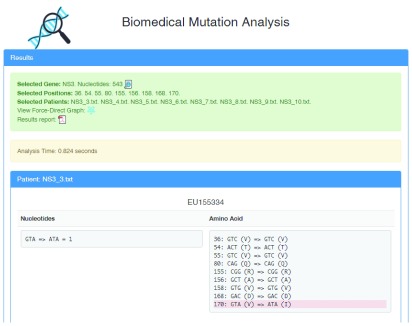
Online textual visualization.

**Figure 5.**
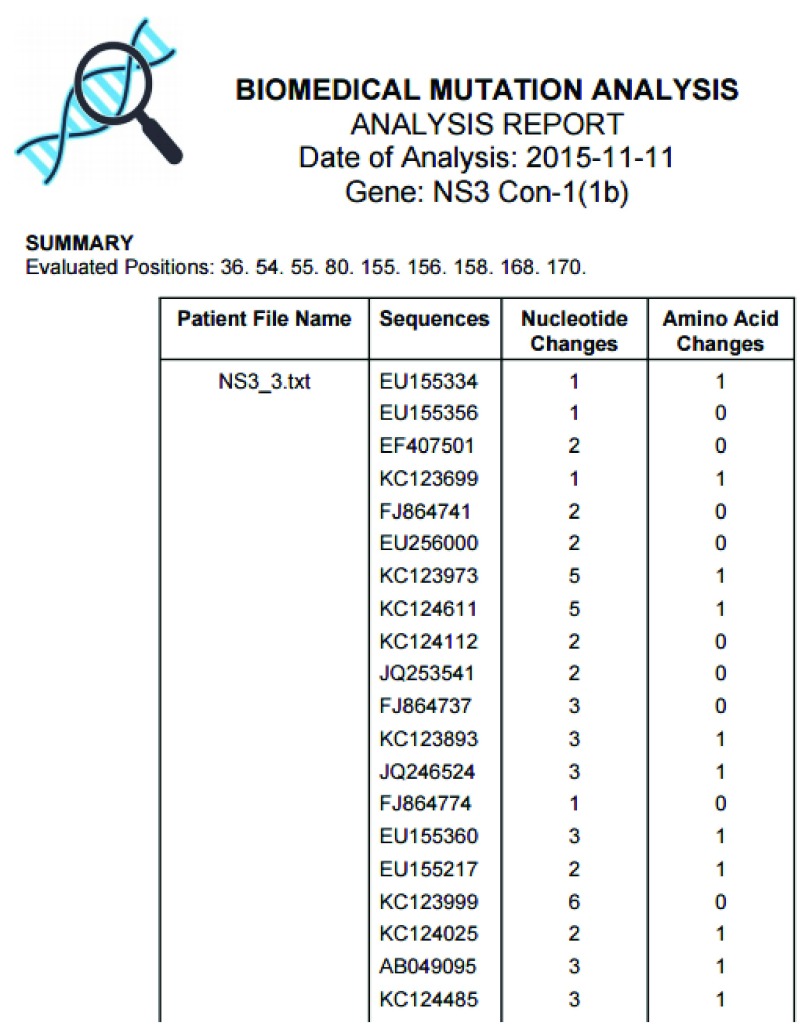
Analysis report.

**Figure 6.**
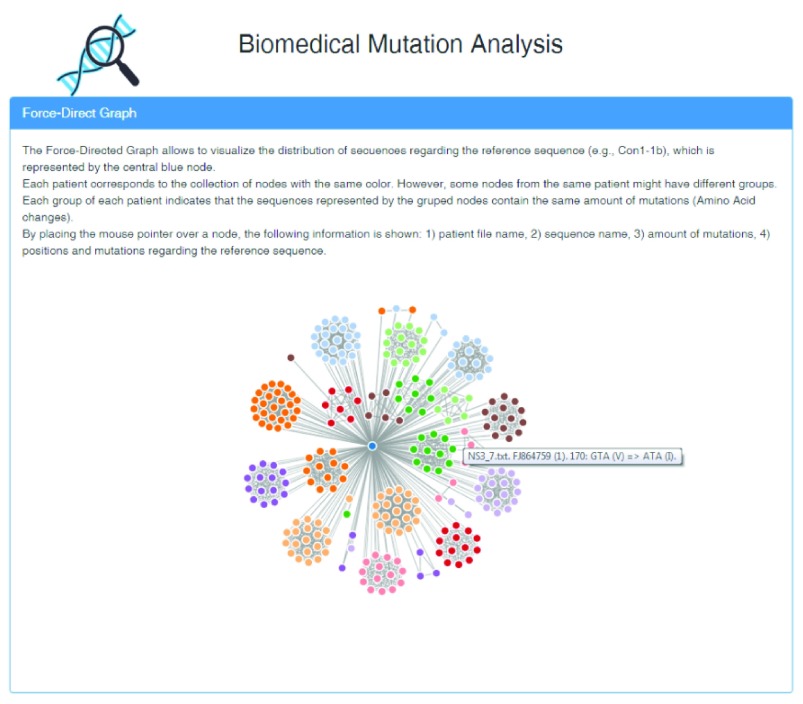
Force-Directed Graph visualization.

The BMA’s core is the analysis algorithm that is able to evaluate multiple patients, where each one can include multiple sequences. In addition, the algorithm can analyze desired positions that the analyst can define. The execution of the algorithm is just one part of the complete analysis process. The analysis process includes the following steps:

1. The analyst accesses BMA via the web site and selects the option “HCV” from the “Mutation Analysis” menu. BMA presents the list of genes available for HCV, which includes the name, description, and reference sequence (by clicking on the corresponding icon). Figure 1 presents the list of available genes.

2. The analyst can use the search icon placed in each gene of the HCV (e.g., NS3, NS5A, NS5B) to proceed to the following step, which corresponds to the selection of the positions to be analyzed. Thus, possible positions are sorted in a list, which includes the number of the position, mutation, antiviral name, inhibitor class, a flag (“Yes” or “No”) that indicates whether or not the position is a main position for the selected gene, and references that can be *in vitro* or *in vivo*. It is important to mention that BMA is flexible allowing the inclusion of further positions, mutations, and antivirals established in new or future research. Regarding references, each position presents the list of academic papers that support scientifically the inclusion of the position in the mutation analysis. Furthermore, for each position, there is an icon that lists the reference details with a link that redirects to one academic search service with the information of the selected reference. Figure 2 presents the list of some positions for the gene NS3.

3. After selecting the positions to perform the analysis, the analyst is asked to provide the patient sequences as plain text files. BMA offers an example dataset for testing the analysis. BMA can automatically read and analyze multiple data files sequentially. These data files may contain a varying number of sequences that represent one patient. BMA can recognize plain text files, which must include the symbol '>' and the sequence name in the first line of the file. The sequence data starts on the second line. Nucleotide data must be written in one line. The sequence must include the symbols: A, C, G, T. Sequences can also include the symbol '-' for specifying missing data. In sequences, blank spaces, tabs, break lines and other symbols are not accepted (see Figure 3).

4. Once patient files are selected, the analysis algorithm is executed. The algorithm presents the results in three different ways:

a) Online textual visualization of necessary nucleotide changes that produce an amino acid change, which generates resistance ( Figure 4).

b) An automatically generated report, which is sent to the analyst’s e-mail address. This report contains a summary of the calculated mutations for each sequence and the full detailed report of the executed analysis ( Figure 5).

c) A “force-directed” graph that identifies mutations of each patient sequence through node grouping, which corresponds to each analyzed sequence. Figure 6 shows that nodes with the same color corresponds to the sequences of a patient. Nodes of a patient are grouped depending on the amount of mutations. In order to know which sequences have mutated, the analyst can place the mouse pointer over a node and the graph presents a popup message with the information of the corresponding node. For instance, Figure 6 also presents a popup message with the information of the node above it. It indicates three facts: 1) the node corresponds to the patient of the file “NS3_7.txt”, 2) the node corresponds to the sequence “FJ864759”, and 3) the sequence has at the position 170 one mutation because in this position the aminoacid should be “GTA(V)”, but it is “ATA(I)”.

For reliable calculations the sequences must contain a substantial part of the genes NS3, NS5A or NS5B (amino acids, aa 1–200).

The analysis algorithm is based on multiple iterations. It collects all patients’ plain text files and iterates in order to analyze all of them independently. For each plain text file, the algorithm collects all sequences. Later on, for each sequence, the algorithm performs a new iteration using the selected positions. Then, for each position, it compares the nucleotide and amino acid of the iterated patient sequence with the reference sequence in the iterated position. At this stage, the information about changes is collected with the corresponding patient, sequence, and position. By finishing the execution of the algorithm, BMA uses the collected results to provide the three aforementioned visualizations.

It is important to mention that BMA cannot align sequences. There are some programs that can do this. For example, CLUSTAL W ^26^ allows multiple alignments. In addition, DNA sequences cannot be edited or manipulated by BMA. No clinical decision should be based only on the result of BMA.

## Conclusions

Software for the detection of mutations associated with resistance to new DAAs is an important tool, because it guarantees accurate and reliable results. Moreover, BMA is freely available, which is different from others such as Bioedit, VectorNTI or MEGA, because it not only allows researchers to perform analysis for the identification of mutations, but also provides detailed information of mutations’ positions, amino acid changes as well as antiviral information and related literature of resistance mutations to the DAA. When BMA is compared with other available tools (e.g., HCV.geno2pheno), it is different because it provides details of the nucleotides changes that produce an amino acid change.

We obtained an online information system “BMA” that was designed and developed, for performing mutation analysis. BMA provides a suitable analysis facilitating all data management. The results can be visualized in a text report as well as graphically.

BMA provides a quick, easy, and effective computer-based analysis of mutations, including complete documentation and examples. Furthermore, the development of different visualization techniques allows for proper interpretation and understanding of the results. The data obtained by BMA will be useful for the assessment and surveillance of HCV resistance to new antivirals, and for the treatment regimens by selecting those DAAs to which the virus is not resistant, avoiding unnecessary treatment failures.

BMA has been designed to be flexible and adaptable. It is a great advantage because it can be used for future evaluation of other viruses such as Influenza and even microorganisms such as bacteria or parasites. Thus, as future work, BMA will analyze a wide range of pathogens. In addition, BMA might be upgraded in order to offer new visualization techniques for facilitating the interpretation of the obtained analysis.

BMA has a small disadvantage. It requires a specific format of sequence information, which is very similar to the FASTA format; thus, the preparation of such information might require a small additional effort. In addition, in future versions, BMA will accept different file formats such as the FASTA format.

## Software availability

Software available from: http://bma.itiud.org

Latest source code: https://github.com/florezfernandez/bma

Archived source code as at the time of publication: http://dx.doi.org/10.5281/zenodo.50994 ^27^

License: GNU General Public License (GPL)
