# Supplementary material for: Biomedical Mutation Analysis (BMA): A software tool for analyzing mutations associated with antiviral resistance
Source: F1000Res. 2016 Aug 2;5:1141. Originally published 2016 Jun 3. [Version 2] doi: 10.12688/f1000research.8740.2 (PMC4984484; doi:10.12688/f1000research.8740.2)
Supplement: Peer review history

# Peer review: Referee response for version 2

**DOI:** 10.5256/f1000research.10090.r15382

**Published:** 2016-08-12

**Authors:** Carla García-Morales

**Affiliations:** 1 Universidad Autónoma del Estado de México, Toluca, Mexico

I have review the new version that the authors of the BMA software have resubmitted. The authors have addressed all my previous comments and I think the tool and the manuscript are appropriate for indexing.

 All the best.

I have read this submission. I believe that I have an appropriate level of expertise to confirm that it is of an acceptable scientific standard.

# Peer review: Referee response for version 1

**DOI:** 10.5256/f1000research.9404.r14899

**Published:** 2016-07-28

**Authors:** Carla García-Morales

**Affiliations:** 1 Universidad Autónoma del Estado de México, Toluca, Mexico

Authors of the BMA tool propose the creation of a bioinformatics tool that identifies changes in nucleotide and amino acid sequences which could represent an impact on antiviral resistance. The suggested system is a promising idea that could be useful for biomedical and pharmacological areas as there is no freely available similar tools.

 One of my concerns, if not the main is English vocabulary and grammar, I would suggest the authors to take advantage of an editing service as some statements could be improved. For a start, from the BMA home page, “In biology, a mutation is a change of the nucleotide sequence of the genome of an org…Mutations play a part in both normal and abnormal biological processes including: evolution, cancer, and the development of the immune system, including junction diversity…Mutation can result in several different types of change in sequences. Mutations in genes can have no effect, alter the product of a gene, or prevent the gene from functioning properly or completely.” I would suggest “In biology, a mutation is a change in the nucleotide sequence on the genome of an org…Mutations play an important role in both normal and abnormal biological processes such as: evolution, cancer, and the development of the immune system, including junction diversity. Mutations can result in several different types of sequence changes; such changes could have no effect, alter the product of the gene, or prevent it from functioning properly or completely.”

 It would also be advisable that authors present a tutorial link in the BMA site.

 As for the manuscript, authors should explain for figure 2 what is shown under the column named “Main”; and again, be careful about expressions such as scientifically support.

 As for any publication on bioinformatics tools, F1000 ask the author to provide sufficient details of codes used for the implementation and operation, unfortunately could not find this information; The only code needed to insert different sequences is the > symbol as in other bioinformatics programs, however the authors should be very clear about this, for example, in the 4th line of number 3 in Results, they only mention “BMA can recognize plain text files, but they have to follow a specific format”, so what do they mean with specific format?, this line is not needed as they explain the required format immediately. Another point that should be improved is in Figure 3. Introduce the patient sequence in a text file format instead of “plain text”

 I would also suggest to change figure 6, for one where the pointer is over the node so that the reader will be able to see what each node represents.

I have read this submission. I believe that I have an appropriate level of expertise to confirm that it is of an acceptable scientific standard, however I have significant reservations, as outlined above.

# Response

**Published:** 2016-07-29

**Authors:** karina salvatierra

**Affiliations:** Universidad Nacional de Misiones, Argentina

Dear Carla Garcia-Morales.

 Thank you so much for your feedback. We want to inform you:

1. We have updated the text in bma.itiud.org as you suggested.
2. In bma.itiud.org, there is in the menu a link called Screencast. It is a video tutorial that presents how to use BMA
3. Information regarding the list of positions has been updating including an explanation of the column “Main”
4. In the section “Software availability”, we provide all details about the source code of the project. Indeed, the project is open source, so everyone can download it.
5. Figure 6 has been also updated in order to present the popup messages of the force directed graph. In addition, the explanation of this figure has been extended.

Thank you so much

 Best Regards

# Peer review: Referee response for version 1

**DOI:** 10.5256/f1000research.9404.r14898

**Published:** 2016-07-15

**Authors:** Vicente Perez-Brocal

**Affiliations:** 1 Public Health and Epidemiology Network Biomedical Research Center (CIBERESP), Madrid, Spain

The article "Biomedical mutation Analysis (BMA): A software tool for analyzing mutations associated with antiviral resistance" describes a new online tool intended for detection of mutations in amino acids implicated in resistance to direct-acting antivirals.

 Despite the complexity and high degree of specifications reflected in the manuscript, especially in the material and methods section, the results section describes each step in a straightforward way that facilitates the implementation of this software. The usage of the language makes this article accessible even for non-experts in the field.

 The methodology has been validated using the nucleotide sequence of some gene from HCV genotype 1b, and a compilation of resistance mutations previously described  in the literature for those genes.

 Minor changes:

- In Materials and Methods section, third paragraph, begins as " The database was design using the tool" and should say "The database was design **ed** using the tool".
- Sentence "For reliable calculations the sequences must contain a substantial part of the genes NS3, NS5A or NS5B" in the Results section results quite vague. How much is a substantial part?
- AAD, that appears in the first paragraph of the conclusions, should be replaced with DAA, for congruence with the rest of the text.

I have read this submission. I believe that I have an appropriate level of expertise to confirm that it is of an acceptable scientific standard.

# Response

**Published:** 2016-07-29

**Authors:** karina salvatierra

**Affiliations:** Universidad Nacional de Misiones, Argentina

Dear Vicente Perez-Brocal

                                  Thank you so much for your feedback.

 Best Regards
